# Characterisation of Cannabis-Based Products Marketed for Medical and Non-Medical Use Purchased in Portugal

**DOI:** 10.3390/molecules29122737

**Published:** 2024-06-08

**Authors:** Bruno Pires, Patrik Oliveira, Ana Y. Simão, João Reis, Sofia Ramos, Ana Paula Duarte, Cláudia Margalho, Tiago Rosado, Mário Barroso, Eugenia Gallardo

**Affiliations:** 1 Centro de Investigação em Ciências da Saúde, Faculdade de Ciências da Saúde da Universidade da Beira Interior (CICS-UBI), 6200-506 Covilhã, Portugal; bruno.mg.pires@gmail.com (B.P.); patrik_99@live.com.pt (P.O.); anaaysa95@gmail.com (A.Y.S.); joao.pedro.reis@ubi.pt (J.R.); sofia.br.2000@gmail.com (S.R.); tiago.rosado@ubi.pt (T.R.); 2 Laboratorio de Fármaco-Toxicologia-UBIMedical, Universidade da Beira Interior, 6200-000 Covilhã, Portugal; 3 Serviço de Química e Toxicologia Forenses, Instituto Nacional de Medicina Legal e Ciências Forenses—Delegação do Centro, 3000-548 Coimbra, Portugal; claudia.i.margalho@inmlcf.mj.pt; 4 Centro Académico Clínico das Beiras (CACB)-Grupo de Problemas Relacionados com Toxicofilias, 6200-000 Covilhã, Portugal; 5 AlphaBiolabs, 14 Webster Court, Carina Park, Warrington WA5 8WD, UK; mario.j.barroso@inmlcf.mj.pt; 6 Serviço de Química e Toxicologia Forenses, Instituto Nacional de Medicina Legal e Ciências Forenses—Delegação do Sul, 1169-201 Lisboa, Portugal

**Keywords:** cannabis, THC, cannabidiol, market products

## Abstract

Cannabis-based products have gained attention in recent years for their perceived therapeutic benefits (with cannabinoids such as THC and CBD) and widespread availability. However, these products often lack accurate labelling regarding their cannabinoid content. Our study, conducted with products available in Portugal, revealed significant discrepancies between label claims and actual cannabinoid compositions. A fully validated method was developed for the characterisation of different products acquired from pharmacies and street shops (beverages, herbal samples, oils, and cosmetic products) using high-performance liquid chromatography coupled with a diode array detector. Linearity ranged from 0.4 to 100 µg/mL (0.04–10 µg/mg) (THC, 8-THC, CBD, CBG, CBDA, CBGA), 0.1–100 µg/mL (0.01–10 µg/mg) (CBN), 0.4–250 µg/mL (0.04–25 µg/mg) (THCA-A), and 0.8–100 µg/mL (0.08–10 µg/mg) (CBCA). Among sampled beverages, none contained detectable cannabinoids, despite suggestive packaging. Similarly, oils often differed from the declared cannabinoid compositions, with some containing significantly higher CBD concentrations than labelled. These inconsistencies raise serious concerns regarding consumer safety and informed decision-making. Moreover, our findings underscore the need for stringent regulation and standardised testing protocols to ensure the accuracy and safety of cannabis-based products.

## 1. Introduction

Cannabis and cannabis-derived products have been present in human societies since ancient times [1]. Cannabinoids are terpene phenolic compounds, with ∆9-tetrahydrocannabinol (THC) being the most psychoactive compound, and as such, it is a controlled substance [2,3,4]. Other than THC, over one hundred other cannabinoids are present in a *Cannabis* plant [5]. One of them is cannabidiol (CBD), a non-psychotropic cannabinoid, and somewhat like THC, it presents diverse pharmacological effects with therapeutic value in the treatment of different medical conditions such as epilepsy, chronic pain, Tourette syndrome, Huntington and Parkinson’s diseases, dyskinesias, and multiple sclerosis [2,4,6]. Since CBD does not present the same addictive properties as THC, as of June 2018, a CBD oral solution (Epidiolex^®^) has been approved by the US Food and Drug Administration (FDA) for the treatment of seizures associated with epilepsy in children [6,7]. In addition to the compounds already mentioned, other main cannabinoids of interest are cannabinol (CBN), cannabigerol (CBG), tetrahydrocannabivarin (THCV), cannabichromene (CBC), tetrahydrocannabinol acid (THCA-A), cannabidiol acid (CBDA), cannabigerol acid (CBGA), tetrahydrocannabivarin acid (THCVA), delta-8 tetrahydrocannabinol (8-THC), cannabidivarin (CBGV), and cannabinovarin (CBNV) [4,6,7]. Cannabis products are constantly evolving and changing, with edibles and vape oils increasing in popularity. Still, smoking of the dried cannabis flower remains the main mode of consumption and administration of this drug [8]. Hemp and marijuana are distinct forms of the plant *Cannabis sativa* L. differing in the quantities of psychoactive compounds, with plants below 0.3% THC production being ranked as hemp and above 0.3% as a drug-type [1,3,9]. While hemp is mostly used to produce industrial products such as fibres and construction materials, marijuana is usually connected to recreational and medicinal use due to its THC and CBD content [1,4,6]. In recent years, the barrier between natural and synthetic cannabinoids has been broken, with new user-tailored substances being created and brought to market [7]. According to the European drug report of 2023 [10], cannabis remains by far the most consumed illicit drug in Europe, with cannabis products becoming increasingly potent each year while maintaining a relatively stable price range [11]. The legalisation of cannabis remains a highly controversial topic, with different countries presenting different control measures when it comes to the possession and consumption of this substances, although its use and legalisation for medical purposes has been increasing [7]. In the USA, public perceptions directly influence cannabis laws, with a medicinal and beneficial ideology constantly growing amongst health professionals and society in general [12]. In 2021, 36 states have already approved laws for the legal use of cannabis in a medicinal context with 18 also approving of its controlled recreational use [12]. Other countries around the world like Canada, Brazil, Italy, Israel, Germany, UK, and the Netherlands also present laws and programs allowing for the medical use of cannabis [12]. In Portugal, according to the law (Law n.º 33/2018 and *Decreto-Lei* n^o^ 8/2019), pharmaceuticals, preparations, and other substances based on cannabis (like Sativex^®^ mouth sprays) are legal as long as they obey the guidelines and prerequisites associated with the cultivation process, preparation, and introduction into the market place [13,14]. Due to the legislation and the easy acquisition of new cannabis-based products (shops, internet, among others), these products have spread enormously and can be found in beverages, herbal samples, oils, cosmetics, etc. However, as has happened in other countries [2,8,15,16,17,18,19], it is unknown whether all these products undergo rigorous control regarding which cannabinoids are present and their concentration. This lack of control may constitute a public health problem, as cannabinoid concentrations can vary in marketed products. The associated risks described include road accidents in the case of driving under the influence of cannabis or when used concomitantly with other substances, such as alcohol. Cannabis is also recurrently associated with cases of occupational accidents, child custody, or drug-facilitated crimes [5].

The aim of this work was to characterise cannabis-based products marketed for medical and non-medical use purchased in Portugal.

## 2. Results and Discussion

### 2.1. Validation Procedure

Several parameters were studied following international guidelines in validation.

#### 2.1.1. Selectivity

**Figure 1:**
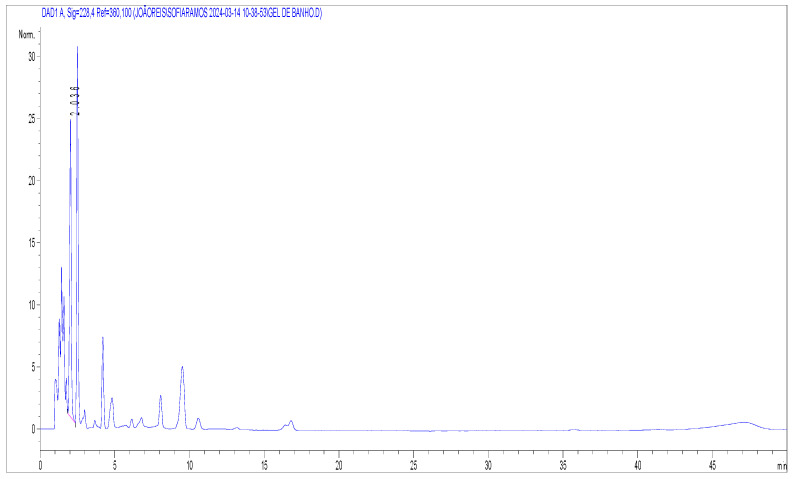
Chromatogram of a blank sample (cosmetic product) with IS. The y-axis corresponds to absorbance, and the x-axis to retention time (min).

Selectivity is defined as the ability to identify and distinguish the target analytes in the presence of other components in the sample, such as metabolites, xenobiotics, concurrently used drugs, decomposition products, or endogenous matrix components. For this study, a blank of reagents (methanol and mobile phase) and blanks of samples of beverages, *Urtica dioica*, oils, and cosmetic products (free of cannabinoids) containing internal standard (Figure 1) were compared with a spiked sample at the lowest limit of quantification (LLOQ) (n = 10). The method was considered selective for the target cannabinoids since the blank of reagents and blank samples revealed no interferences at the retention times of the analytes in study. The blank of reagents and blank of samples revealed signals lower than 15% compared to the signal of the analytes, and a signal lower than 5% compared with the internal standard one (200 µL at 1 mg/mL for cosmetic products and herbal extracts, and 10 µL at 100 µg/mL for beverages and oils). The analytes appear well separated with no interferences.

#### 2.1.2. Calibration Curves and Limits

The linearity study (n = 5) was evaluated by using increasing concentrations of each target cannabinoid. As acceptance criteria, the measured concentrations should present a mean relative error (RE) within ±15% of the theoretical concentration, except for the LLOQ where a ±20% interval is accepted. For each analyte, calibrators were evenly distributed across a concentration range of 0.4 to 100 μg/mL (0.04–10 µg/mg) for most compounds under study, except for CBN (0.1 to 100 μg/mL; 0.01–10 µg/mg), THCA-A (0.4 to 250 μg/mL; 0.04–25 µg/mg), and CBCA (0.8 to 100 μg/mL; 0.08–10 µg/mg). The LLOQ was considered the minimum concentration of the target cannabinoids that could be determined with a precision, measured through coefficient of variation (CV%) ≤ 20%, and an accuracy measured through RE within ±20% of the nominal concentration. The LLOQs were 0.1 µg/mL (0.01 µg/mg) for CBN, 0.8 µg/mL (0.08 µg/mg) for CBCA, and 0.4 µg/mL (0.04 µg/mg) for the remaining compounds. The limit of detection (LOD) was not systematically assayed and was hence considered equal to that of the LLOQ. Table 1 summarises the linearity and limits data.

The LLOQs obtained can be considered quite good compared with other authors who used similar extraction solvents and HPLC-UV for cannabinoids separation and detection. The method described herein achieved lower LLOQs than those reported by Aubin et al. [20]. The authors proposed an analytical method to determine 16 cannabinoids in flowers and extracts and achieved values of 4 µg/mL. Also, Mudge et al. [21] proposed another analytical method to determine nine cannabinoids in *Cannabis sativa* dried flowers and oils, achieving LLOQs of 0.5 µg/mL for CBN, 1 µg/mL for THC and CBD, and 5 µg/mL for THCA-A and CBDA. Overall, these authors’ LLOQs were greater than ours, so we can assume the method described herein presents greater sensitivity for the selected cannabinoids. Nonetheless, and although HPLC-UV is the most commonly described analytical technique for these analytes, some authors propose methods with gas chromatography coupled to mass spectrometry (GC-MS). Fernández et al. [2] proposed a GC-MS method to determine THC, CBN and CBD in Argentinean commercially available preparations. The authors reveal LLOQs of 0.1 µg/mL for THC and CBD, and 0.04 µg/mL for CBN. These LLOQs are lower than those we report, and MS detection sensitivity combined with diethyl ether use for sample clean up could have contributed for this achievement. It should be noted that these authors reveal sample preparation recoveries of 95 to 103% while ours range from 61 to 99%. Moreover, the herein described method is more ambitious since allows the determination of nine cannabinoids. Another method that used GC-MS detection and diethyl ether for sample preparation was the one proposed by Franzin et al. [22]. These authors aimed to determine seven cannabinoids in therapeutic preparations from an Italian region, reporting LLOQs of 0.2 µg/mL for all of them. The method described herein is only surpassed by CBN, in which a LLOQ of 0.1 µg/mL (0.01 µg/mg) was obtained. Once again, GC-MS detection and diethyl ether use for sample preparation might have helped to increase sensitivity in these authors’ methods.

#### 2.1.3. Intermediate, Intra-, and Inter-Day Precision and Accuracy

Over the course of five days, inter-day precision and accuracy were assessed at nine concentrations (0.4, 0.8. 1.6, 3.1, 6.3, 12.5, 25, 50 and 100 µg/mL; 0.04, 0.08, 0.16, 0.31, 0.63, 1.25, 2.5, 5 and 10 µg/mg) for most of the target cannabinoids. Regarding CBN, and since a lower limit of quantification was achieved, 11 calibrators were adopted by adding 0.1 and 0.2 µg/mL (0.01 and 0.02 µg/mg) concentrations. Concerning THCA-A, the calibration range was extended in the upper limit and a concentration of 250 µg/mL (25 µg/mg) was added. Lastly, eight calibrators were adopted for CBCA over the range between 0.8 (LLOQ) and 100 µg/mL (0.08–10 µg/mg). In this assay, the obtained CVs were typically lower than 13.16% for all analytes and calibrators, with exception of THCA-A that revealed a CV of 17.02% at the LLOQ calibrator (0.4 µg/mL; 0.04 µg/mg). Moreover, the maximum observed inaccuracy (RE) for all cannabinoids and evaluated concentrations was within ±13.33%.

Six replicates of samples spiked with the target analytes at four concentration levels (LLOQ, 1, 50 and 100 µg/mL; LLOQ, 0.1, 5, 10 µg/mg), which were analysed on the same day to assess the intra-day precision and accuracy. For CBN and THCA-A, the intra-day precision and accuracy was also evaluated at 0.4 and 250 µg/mL (0.04 and 25 µg/mg), respectively. Overall, the intra-day precision assay revealed CVs typically lower than 12.22%, while a maximum inaccuracy was found to be within ±14.50% for all target analytes.

Additionally, intermediate precision and accuracy were assessed. Also, over the course of five distinct days, four quality control samples (QCs) were evaluated. The adopted QCs concentrations were the LLOQ, 1, 10, and 100 µg/mL (LLOQ, 0.1, 1, and 10 µg/mg) (n = 3) for each daily run for most cannabinoids. Once again, and regarding CBN and THCA-A, intermediate precision and accuracy were also assessed for 0.4 and 250 µg/mL (0.04 and 25 µg/mg), respectively. Intermediate precision revealed a maximum CV of 14.70% for all target analytes, with the exception of THCA-A, which revealed a CV of 15.51%, again at the LLOQ calibrator. The maximum RE was proven to be within a ±14.60% for all cannabinoids. This way, the herein described evaluation confirms that the analytical method is precise and accurate for the determination of the target analytes. All data are shown on Supplementary Table S1.

#### 2.1.4. Recovery

The recovery is the percentage relationship between the response obtained by the detector in the analysis for the amount of analyte added and extracted from the matrix and the response obtained for the amount of analyte theoretically present in the matrix. In the analytical procedure described herein, extraction was performed for herbal and cosmetic products. In order to assess the recovery of the target cannabinoids present in the samples, a comparison was performed between the areas of the chromatographic peaks of two sample sets: one that was fortified (flowers of *U. dioica* and a cosmetic product free of cannabinoids) with the target cannabinoids before going through the extraction process, and another that was extracted (flowers of *U. dioica* and a cosmetic product free of cannabinoids) with analytes that were added to the obtained extract after the extraction procedure. In both cases, the internal standard was added after the extraction process. As per the protocol outlined [21], this parameter was assessed with *U. dioica* flowers since they mimic cannabis flowers and herbal samples (teas) with the advantage of not revealing cannabinoids on its composition. In the case of beverages and oils, since linearity was evaluated by diluting the standards of each compound, recovery was not calculated.

Table 2 presents the recovery data obtained for each cannabinoid. This parameter was evaluated by fortifying the sets of samples at three concentration levels (LLOQ, 50 and 100 µg/mL; LLOQ, 5 and 10 µg/mg) for most target analytes. Concerning CBN and THCA-A, recoveries were also assessed for 0.4 and 250 µg/mL (0.04 and 25 µg/mg), respectively. Each of these concentrations was analysed in triplicate. Overall, the obtained recoveries were considered more than acceptable with values ranging from 69 to 99% for CBD, 73 to 93% for CBDA, 76 to 99% for CBG, 61 to 97% for CBN, 64 to 95% for CBGA, 74 to 91% for THC, 63 to 90% for 8-THC, 77 to 89% for THCA-A, and 67 to 87% for CBCA.

One should keep in mind that according with FDA and EMA guidelines [23,24] the obtained recovery values do not necessarily have to be 100% to be accepted, as long as they demonstrate reproducibility, precision and consistency.

Nonetheless, the extraction efficiencies reported in this method can be considered slightly lower when compared with those achieved by other authors with same goal. Mudge et al. [21] report recoveries of 98 to 108% for CBDA and 98 to 104% for THC, not evaluating the recoveries for the remaining compounds due to cost of the reference standards. In the method proposed herein, the recoveries were 73–93% for CBDA and 74–91% for THC. For the remaining analytes the extraction efficiencies slightly decreased but no comparison is possible since Mudge et al. [21] did not evaluate those. The authors used 80% methanol for sample preparation which might justify the differences obtained. Greater recoveries were also obtained by Fernández et al.’s [2] method. These authors reveal extraction efficiencies of 103% for CBD, 100% for THC and 95% for CBN, while ours were 69–99% for CBD, 74–91% for THC and 61–97% for CBN. No other cannabinoids were determined in the Fernández et al. [2] method, and, once again, the use of a different sample preparation approach might have improved their recoveries. These authors used diethyl ether as a solvent. Both Aubin et al. [20] and Franzin et al. [22] with similar works to the one that we present, have not reported recovery data.

It is important to note that the developed method is simple, easy, and suitable for its application in routine dosage of these compounds, as pharmacopeias (e.g., German Pharmacopoeia (Official Part, 24.4.2018 B5, published in 24 April 2018)) indicate that the recommended method is HPLC-DAD.

### 2.2. Characterisation of Cannabis-Based Products

Different products were acquired from street shops and pharmacies for analysis, including 6 beverages (3 beers, 2 ice teas, and 1 carbonated beverage), 10 oils, 6 herbal samples, and 9 semi-solid forms (cosmetic products) (Table 3).

The results obtained for the composition and cannabinoid content in the samples have shown that most of the studied products was mislabelled. In the six beverages analysed (three beers, two ice teas, and one carbonated beverage), no cannabinoids were detected. Upon careful examination of the labels, the manufacturer did not describe the presence of *Cannabis sativa* extract. However, the packaging’s imagery is very appealing, with the word cannabis mentioned several times as well as allusive images that imply to the consumer that it is a cannabis-based beverage.

**Figure 2:**
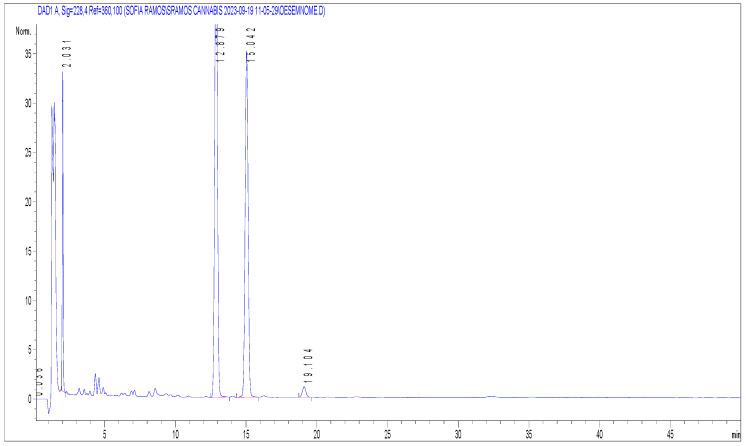
Chromatogram obtained after the HPLC-DAD analysis of oil #1. The y-axis corresponds to absorbance, and the x-axis to retention time (min). Ketoprofen (2.03 min); CBD (12.88 min); CBG (15.04 min); CBN (19.10 min).

Concerning the oils, the expected composition according to the product manufacturer for sample oil #4 was 20% CBD, and it also claimed to contain other cannabinoids (CBN, CBC, CBDA, CBG, CBGA, and CBCA). However, the results obtained were 16.13% CBD, and no other cannabinoids were detected. In sample oil #1 (Figure 2), compounds CBD, CBG, and CBN were detected, with concentrations of 7.50%, 5.80%, and 0.06%, respectively. The expected concentrations according to the label were 5% CBG and 5% CBN, of which CBN was not observed. The sample contained a significantly higher concentration of CBD than labelled, while CBN was mislabelled. Concerning CBG, the measured concentration is lower than expected, taking into account a variation criterion of 10% used in the US Pharmacopeia [25]. The same occurred with oil #10, where the detected concentration of CBD was 5.02%, while the label indicates a concentration of 10%. Regarding sample oil #3, a concentration of 10% CBD was expected according to the manufacturer’s label. However, a concentration of 1.82% CBD was found. The labels of sample oil #2, oil #5, oil #6, and oil #7 did not provide detailed information about composition and concentration. However, all of them mentioned CBD on the packaging, except for sample oil #5, which only claimed to contain hemp. Therefore, as they were marketed as cannabinoid oils, a significant concentration of CBD was expected. Cannabinoids were not detected in sample oils #2, #7, or #8. In sample oil #5, 0.08% of CBN was quantified. In sample oil #6, the detected concentrations were 5.00% for CBD, 0.08% for CBG, and 0.05% for THCA-A. Finally, in oil #9, CBD (0.29%) and THCA-A (0.07%) were detected, while the label only mentioned the presence of CBD.

Concerning herbal samples, they were only stated to have a THC composition of less than 0.2% (in compliance with regulations). In all of these samples, this premise was confirmed. Several cannabinoids were detected, however, in very small quantities. Regarding cosmetic products, none of them made mention of their composition regarding the present cannabinoids except cream 3. In this latter case, the detected concentration of CBD is as indicated by the manufacturer (CBD < 1%).

These products, whose labels do not provide detailed information, raise significant concerns as they prevent consumers from making informed choices about the type of product they want to consume and the benefits they want to achieve with it.

There are other published studies on this topic in other countries. Mouton et al. [15] examined forty commercially available products, conducting quantitative analyses across a varied spectrum of consumer goods, encompassing soft drinks, honey, coffee, oils, gummy bears, chocolate, and other items purported to contain CBD. Their objective was to assess the consistency between the actual CBD content of these products and the values indicated in the labels. The products, sourced from South Africa, the USA, Spain, the Netherlands, and Indonesia, spanned various international markets. Through their investigation, the authors uncovered significant labelling discrepancies: numerous products were either over- or under-labelled, some lacked CBD content disclosure altogether, while others contained undetectable CBD levels. Among the 16 analysed oils, only 3 fell within the declared range, with 8 products being over-labelled and 1 under-labelled. Additionally, three products failed to specify the amount of CBD and showed no detectable CBD content. Notably, among the five topical products examined, product 18 stood out, displaying a CBD content 98.29% lower than stated on the packaging. Furthermore, product 20, purportedly containing 10 g of CBD hemp oil, exhibited no detectable CBD. In the case of drinks and other products, all were over-labelled except for product 34, marketed as “water-soluble CBD sachets”. This shows that mislabelling extends beyond quantity, including misrepresentations of CBD’s chemical properties, such as marketing water-soluble sachets despite CBD’s insolubility in water. Wiley et al. [16] conducted a comprehensive analysis into the scientific, marketing, and legal dimensions of CBD, revealing incredible insights regarding market assertions. CBD is often marketed as a dietary supplement, food additive, cosmetic ingredient, or drug, with each classification subject to distinct FDA regulations. Drug approval mandates rigorous scrutiny of safety and efficacy, contrasting with minimum required post-market safety for dietary supplements, cosmetics, and food/beverages. The absence of a robust regulatory framework surrounding CBD’s sale and legal status has fuelled the proliferation of CBD products, allowing marketers to navigate the regulatory landscape while capitalising on public enthusiasm for CBD’s potential therapeutic benefits. The authors addressed conflating scientific findings on THC or cannabis effects, which contain THC alongside CBD and other cannabinoids, with CBD’s isolated effects. In addition to the false therapeutic claims surrounding CBD, inaccurate labelling continues to be a problem, as evidenced by studies revealing significant disparities between labelled and independently verified CBD concentrations. Despite claims of laboratory testing for CBD content by some companies, validation remains elusive due to CBD’s exclusion from the United States Pharmacopeia. Even in products with verified CBD content, concentrations may fall short of yielding desired pharmacological effects, particularly via oral consumption. Bonn-Miller et al. [17] undertook a similar investigation to that of Mouton et al. [15], opting to source products from online markets to enhance sample diversity and ensure the representation of available products. Analysing approximately 84 products, their findings were unsurprising: concerning CBD labelling, 42.85% of products were under-labelled, 26.19% were over-labelled, and only 30.95% were accurately labelled. Additionally, analysis of other cannabinoids revealed generally low and negligible concentrations, yet the psychoactive compound THC was detected in nearly 22% of the samples. Likewise, a study to assess the quality of 14 CBD oils commercially available in European countries, incorporating chemical profiling of cannabinoids, terpenes and oxidation products, was conducted by Pavlovic et al. [18]. As anticipated, for CDB and given the preceding regulatory gaps, 9 out of the 14 samples exhibited concentrations significantly divergent from the declared amounts, while the remaining 5 maintained the levels within acceptable limits. Oils 8 and 10 displayed CBD levels higher than those specified by producers, whereas oils 3 and 14 presented significantly lower values than stated. Additionally, the study successfully quantified the six most significant cannabinoids: THC, THCA-A, CBDA, CBN, CBG, and CBGA [18]. Interestingly, 12 out of 14 samples contained THC, although presenting with high variability, predominantly showed low levels (0.2%). Oil 6 was the only that contained a notable amount of THC (0.35%), raising concerns as the manufacturer claimed the product to be THC-free. CBN was also quantifiable in most samples, except for oil 14. Edible cannabis products, also lacking regulation, continue to fall short of meeting basic label accuracy standards akin to pharmaceuticals products. Vandrey et al. [19] substantiated this assertion by analysing 75 edible products from three major metropolitan areas in the USA. Their results revealed that only 17% of products were accurately labelled, with 23% under-labelled and a staggering 60% over-labelled in terms of THC content. CBD was no different and while 44 products contained detectable levels of CBD, only 13 were appropriately labelled for CBD content. More than half of the evaluated products exhibited significantly lower cannabinoid content than indicated, with some containing negligible THC amounts. Conversely, certain products contained notably higher THC levels than labelled, posing risks of adverse effects for consumers. Fernández et al. [2] employed a GC-MS approach to quantify cannabinoids in cannabis oils originating from Argentina. They developed, validated, and applied a method for the main cannabinoids THC, CBD, and CBN, while also conducting a pre-screening in full scan mode for THC, CBD, CBN, CBC, CBG, THCA-A, and CBDA. Among the 10 oils analysed, oils 1 and 2 (from controlled/regulated production) exhibited CBD levels consistent with product labelling and low THC levels, as expected from hemp-derived oils. In contrast, cannabis oils from uncontrolled/unregulated production displayed notably different concentrations of CBD, THC, and CBN. Oils 3 and 7 demonstrated high levels of THC and low or undetectable concentrations of CBD, while oils 6, 8, and 9 showed undetectable CBD content and THC levels ranging from 1.3 to 4.3 mg/mL. These results align with previous findings, indicating a recurring trend in cannabinoid concentrations across various studies.

All these findings, as well as those presented in this article, highlight the urgent need for rigorous testing and quality control measures in the realm of cannabis products.

## 3. Materials and Methods

### 3.1. Reagents and Standards

Analytical standards of ∆9-tetrahydrocannabinolic acid (THCA-A), ∆8-tetrahydrocannabinol (8-THC), cannabinol (CBN), cannabidiol (CBD), cannabidiolic acid (CBDA), cannabigerol (CBG), cannabigerolic acid (CBGA), cannabichromenic acid (CBCA), ∆9-tetrahydrocannabinol (THC), and ketoprofen (internal standard; IS) were obtained from Sigma-Aldrich S.A. (Lisboa, Portugal). Trifluoracetic acid, ethanol, acetonitrile, and methanol (HPLC-grade) were obtained from Enzymatic (Santo Antão do Tojal, Portugal).

Working solutions were prepared in methanol a concentration of 1 mg/mL and 100 µg/mL for all analytes. The prepared standards were stored at 4 °C, protected from light until use.

### 3.2. Chromatographic Analysis

**Figure 3:**
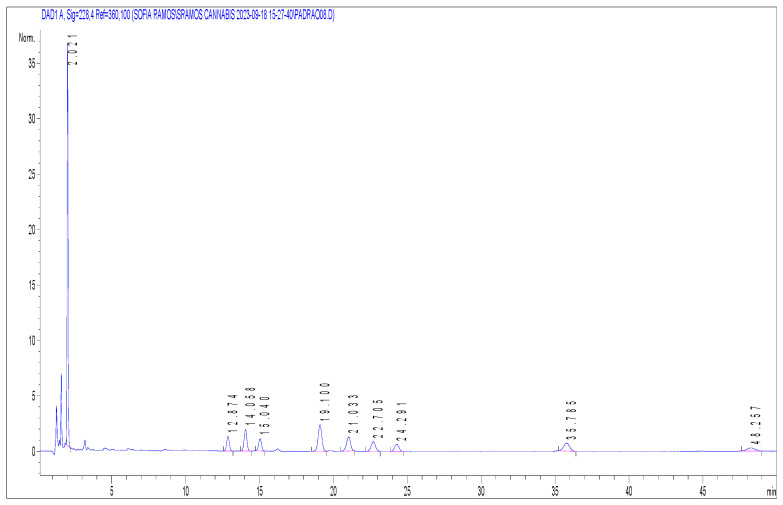
Chromatogram at 0.8 µg/mL of all cannabinoids. The y-axis corresponds to absorbance, and the x-axis to retention time (min). Ketoprofen (2.02 min); CBD (12.87 min); CBDA (14.06 min); CBG (15.04 min); CBN (19.10 min); CBGA (21.03 min); THC (22.71 min); 8-THC (24.29 min); THCA-A (35.79 min); CBCA (48.26 min).

For the analysis, a high-performance liquid chromatography coupled with a photodiode array detector (HPLC-DAD) was employed (model 1290 Agilent Technologies, Soquímica, Lisbon, Portugal). The chromatographic column utilised was a reverse-phase Cortes Shield RP18 4.6 × 150 mm, 2.7-micron (Waters Portugal, Lisboa, Portugal). The HPLC was operated in isocratic mode, with a mobile phase comprised of 0.1% trifluoracetic acid in water with acetonitrile (41:59; *v*/*v*), with a flow of 1 mL/min. Column temperature was kept at 35 °C and the sampler at 4 °C. The injection volume was 20 µL. The chosen wavelength for the diode array detection was 228 nm [20]. Under these conditions the retention times obtained are summarised in Table 4. As example, Figure 3 shows a chromatogram of a sample spiked at 0.8 µg/mL for all target cannabinoids.

### 3.3. Sample Preparation

Different commercial samples (31 samples) of cannabis-based products (herbal samples (teas), beverages, creams, oils) obtained from street shops (big and small supermarkets, hemp shops) and pharmacies were analysed.

Herbal extracts were grounded to a fine powder to ensure sample homogeneity. For the extraction of solid and semi-solid samples (herbal and cosmetic creams), 0.5 g of sample was weighed and agitated for 15 min with 20 mL of acetonitrile, and 200 µL of IS at a concentration of 1 mg/mL was added. The mixture was then centrifuged at 2579 g for 15 min at 4 °C, and the clear supernatant was recovered and filtered through a cellulose membrane filter with 0.22 µm pore size. 100 µL of the filtrate was transferred to a vial.

For the oils, a dilution of 1:1000 was made. A total of 50 µL of the oil samples were taken, transferred to a 50 mL volumetric flask, and the volume was completed with pure ethanol. A total of 70 µL of this flask was transferred to a vial, and 20 µL of acetonitrile and 10 µL of IS were added.

In the case of beverages, about 3 mL of the sample was filtered through a cellulose membrane filter with 0.22 µm pore size. Subsequently, 90 µL of the filtered was transferred to a vial with the addition of 10 µL of IS.

## 4. Conclusions

This paper describes the validation of an analytical method to determine nine cannabinoids by liquid chromatography–diode array detector. The developed methodology was fully validated according to internationally accepted guidelines, and good precision and accuracy were obtained. In this study, 31 samples of over-the-counter cannabis-based products bought in Portugal were analysed. Significant discrepancies in labelling accuracy across a wide variety of products are evident. In most of the analysed samples, the concentrations were found to be below what was indicated on the label. Additionally, many of these products did not contain information about which cannabinoids were present and their concentrations. However, the packaging implied that they were cannabis-containing products since this word was mentioned. There is an urgent need for standardised regulations and robust quality assurance protocols. Beyond mere labelling inaccuracies, the implementation of quality control mechanisms allows the safeguarding of consumer safety and ensuring product efficacy within the burgeoning cannabis industry.

## Figures and Tables

**Table 1:** Linearity data (n = 5).

Analyte	Product	Weight	Linear Range (µg/mL) (µg/mg for semisolid and solid samples)	Linearity		R^2^ *	LLOQ (µg/mL) (µg/mg for semisolid and solid Samples)
				Slope *	Intercept *		
CBD	Beverage/oil	1/x	0.4–100	0.1206 ± 0.0283	−0.0058 ± 0.0065	0.9990 ± 0.0004	0.4
	Herbal sample	1/x	0.04–10	0.0161 ± 0.0008	0.0011 ± 0.00137	0.9953 ± 0.0017	0.04
	Cosmetic product	1/x	0.04–10	0.0223 ± 0.0070	0.0009 ± 0.00168	0.9943 ± 0.0038	0.04
CBDA	Beverage/oil	1/x	0.4–100	0.1952 ± 0.0364	−0.0110 ± 0.0115	0.9986 ± 0.0004	0.4
	Herbal sample	1/x	0.04–10	0.0256 ± 0.0008	−0.0001 ± 0.0029	0.9974 ± 0.0016	0.04
	Cosmetic product	1/x	0.04–10	0.0366 ± 0.0012	0.0030 ± 0.0049	0.9959 ± 0.0025	0.04
CBG	Beverage/oil	1/x	0.4–100	0.1198 ± 0.0315	−0.0076 ± 0.0073	0.9979 ± 0.0020	0.4
	Herbal sample	1/x	0.04–10	0.0165 ± 0.0022	0.0101 ± 0.0182	0.9953 ± 0.0040	0.04
	Cosmetic product	1/x	0.04–10	0.0244 ± 0.0037	−0.0025 ± 0.0063	0.9941 ± 0.0032	0.04
CBN	Beverage/oil	1/x	0.1–100	0.2778 ± 0.0738	−0.0059 ± 0.0099	0.9984 ± 0.0007	0.1
	Herbal sample	1/x	0.01–10	0.0353 ± 0.0049	−0.0296 ± 0.0535	0.9937 ± 0.0026	0.01
	Cosmetic product	1/x	0.01–10	0.0438 ± 0.0042	−0.0291 ± 0.0527	0.9928 ± 0.0013	0.01
CBGA	Beverage/oil	1/x	0.4–100	0.1986 ± 0.0355	−0.0142 ± 0.0134	0.9987 ± 0.0003	0.4
	Herbal sample	1/x	0.04–10	0.0265 ± 0.0040	0.0009 ± 0.0022	0.9945 ± 0.0037	0.04
	Cosmetic product	1/x	0.04–10	0.0374 ± 0.0011	−0.0280 ± 0.0573	0.0035 ± 0.0033	0.04
THC	Beverage/oil	1/x	0.4–100	0.1119 ± 0.0294	−0.0059 ± 0.0104	0.9988 ± 0.0004	0.4
	Herbal sample	1/x	0.04–10	0.0171 ± 0.0019	−0.0310 ± 0.0556	0.9975 ± 0.0005	0.04
	Cosmetic product	1/x	0.04–10	0.0241 ± 0.0031	−0.0023 ± 0.0054	0.9961 ± 0.0019	0.04
8-THC	Beverage/oil	1/x	0.4–100	0.0975 ± 0.0244	−0.0060 ± 0.0052	0.9989 ± 0.0004	0.4
	Herbal sample	1/x	0.04–10	0.0151 ± 0.0019	−0.0309 ± 0.0561	0.9963 ± 0.0021	0.04
	Cosmetic product	1/x	0.04–10	0.0178 ± 0.0020	−0.0053 ± 0.0117	0.9960 ± 0.0017	0.04
THCA-A	Beverage/oil	1/x	0.4–250	0.1738 ± 0.0267	0.0036 ± 0.0358	0.9967 ± 0.0021	0.4
	Herbal sample	1/x	0.04–25	0.0195 ± 0.0034	−0.0315 ± 0.0556	0.9930 ± 0.0200	0.04
	Cosmetic product	1/x	0.04–25	0.0326 ± 0.0030	0.0029 ± 0.0060	0.9939 ± 0.0044	0.04
CBCA	Beverage/oil	1/x	0.8–100	0.0684 ± 0.0143	−0.0092 ± 0.0089	0.9987 ± 0.0005	0.8
	Herbal sample	1/x	0.08–10	0.0102 ± 0.0014	−0.0307 ± 0.0562	0.9953 ± 0.038	0.08
	Cosmetic product	1/x	0.08–10	0.0141 ± 0.0016	−0.0316 ± 0.0582	0.9940 ± 0.0044	0.08

* Mean values ± standard deviation. ∆8-tetrahydrocannabinol (8-THC), ∆9-tetrahydrocannabinol (THC), ∆9-tetrahydrocannabinolic acid (THCA-A), cannabichromenic acid (CBCA), cannabidiol (CBD), cannabidiolic acid (CBDA), cannabigerol (CBG), cannabigerolic acid (CBGA) and cannabinol (CBN).

**Table 2:** Study of the recovery (%) at three concentration levels; (n = 3).

Product	Analyte	Concentration (µg/mg)					
*U. dioica*		0.01 *	0.04 *	0.08 *	5 *	10 *	25 *
	CBD		69.10 ± 0.70		77.87 ± 1.08	91.29 ± 0.84	
	CBDA		73.46 ± 8.26		72.74 ± 0.59	92.68 ± 0.65	
	CBG		82.97 ± 10.48		75.50 ± 1.50	99.47 ± 3.90	
	CBN	76.98 ± 0.96	61.37 ± 2.52		76.46 ± 0.84	87.35 ± 0.82	
	CBGA		64.39 ± 1.36		74.82 ± 1.53	87.82 ± 1.88	
	THC		74.15 ± 6.67		87.02 ± 2.49	90.90 ± 2.32	
	8-THC		62.54 ± 2.17		75.72 ± 2.75	83.64 ± 2.27	
	THCA-A		84.43 ± 3.50		86.10 ± 3.76	86.48 ± 0.78	80.58 ± 1.20
	CBCA			67.10 ± 3.45	75.01 ± 1.49	83.08 ± 0.30	
Cosmetic products		0.01 *	0.04 *	0.08 *	5 *	10 *	25 *
	CBD		91.76 ± 4.71		93.93 ±4.22	98.82 ± 9.17	
	CBDA		87.37 ± 2.89		91.05 ± 0.35	92.80 ± 3.54	
	CBG		77.93 ± 0.61		81.40 ± 2.69	91.95 ± 3.61	
	CBN	72.28 ± 4.89	75.74 ± 5.04		96.76 ± 2.23	96.76 ± 0.53	
	CBGA		78.55 ± 4.17		82.50 ± 2.55	95.35 ± 1.63	
	THC		80.35 ± 4.03		83.65 ± 0.92	87.40 ± 3.96	
	8-THC		83.55 ± 8.41		89.85 ± 2.05	87.75 ± 4.88	
	THCA-A		76.85 ± 0.64		84.55 ± 7.14	86.14 ± 4.12	88.75 ± 0.92
	CBCA			77.50 ± 5.80	77.25 ± 3.40	86.35 ± 2.90	

* Mean values ± standard deviation. ∆8-tetrahydrocannabinol (8-THC), ∆9-tetrahydrocannabinol (THC), ∆9-tetrahydrocannabinolic acid (THCA-A), cannabichromenic acid (CBCA), cannabidiol (CBD), cannabidiolic acid (CBDA), cannabigerol (CBG), cannabigerolic acid (CBGA) and cannabinol (CBN).

**Table 3:** Concentrations found in commercial products.

Oils			
	Compound	Concentration	Concentration Found on the Label
#1	CBD	7.50%	CBG 5% and CBN 5%
	CBG	5.80%	
	CBN	0.06%	
#2	n.d.	n.d.	Did not present information
#3	CBD	1.82%	CBD 10% and <0.2% THC
#4	CBD	16.13%	CBD 20%; CBN, CBC, CBDA, CBG, CBGA and CBCA
#5	CBN	0.08%	Did not present information
#6	CBD	5.00%	Did not present information
	CBG	0.08%	
	THCA-A	0.05%	
#7	n.d.	n.d.	Did not present information
#8	n.d.	n.d.	<0.2% THC
#9	CBD	0.29%	No concentration described (only information: CBD)
	THCA-A	0.07%	
#10	CBD	5.02%	CBD 10%
**Herbal products**			
#1	CBD	0.01%	No concentration described (only information: <0.2% THC)
	CBDA	0.01%	
#2	CBD	0.23%	No concentration described (only information: <0.2% THC)
	CBDA	0.23%	
	CBG	0.02%	
	CBN	3 × 10^−3^%	
	CBGA	0.01%	
	THC	0.01%	
	CBCA	0.01%	
#3	CBD	0.26%	No concentration described (only information: <0.2% THC)
	CBDA	0.31%	
	CBG	0.02%	
	CBN	3 × 10^−3^%	
	CBGA	0.01%	
	THC	0.01%	
	CBCA	0.01%	
#4	CBD	0.10%	No concentration described (only information: <0.2% THC)
	CBDA	0.10%	
	CBG	0.01%	
	CBGA	3 × 10^−3^%	
	THC	3 × 10^−3^%	
#5	CBD	0.07%	No concentration described (only information: <0.2% THC)
	CBDA	0.07%	
	CBG	4 × 10^−3^%	
	CBGA	3 × 10^−3^%	
	THC	1 × 10^−3^%	
#6	CBD	0.03%	No concentration described (only information: <0.2% THC)
	CBDA	0.03%	
	CBG	3 × 10^−3^%	
**Cosmetic products**			
Cream #1	CBD	0.24%	No concentration described (only information: CBD)
	CBG	3 × 10^−3^%	
Cream #2	CBD	0.01%	No concentration described (only information: CBD)
	CBG	3 × 10^−3^%	
Cream #3	CBD	0.01%	No concentration described (only information: CBD < 1%)
	THC	0.05%	
Shower gel #4	n.d.	n.d.	*Cannabis sativa* seed oil
Shampoo #5	CBD	4 × 10^−3^%	*Cannabis sativa* seed oil, *Cannabis sativa* seed extract
Balm # 6	CBD	0.09%	No concentration described (only information: CBD)
Cream #7	n.d.	n.d.	*Cannabis sativa* (Hemp) seed oil
Cream #8	CBD	0.01%	No concentration described (only information: CBD)
Cream #9	n.d.	n.d	*Cannabis sativa* seed oil

n.d. Not detected. ∆9-tetrahydrocannabinol (THC), ∆9-tetrahydrocannabinolic acid (THCA-A), cannabichromenic acid (CBCA), cannabidiol (CBD), cannabidiolic acid (CBDA), cannabigerol (CBG), cannabigerolic acid (CBGA) and cannabinol (CBN).

**Table 4:** Retention times for the identification of the target analytes.

Analyte	Retention Time (min)
KET *	2.02
CBD	12.87
CBDA	14.06
CBG	15.04
CBN	19.10
CBGA	21.03
THC	22.71
8-THC	24.29
THCA-A	35.79
CBCA	48.26

* Internal Standard. ∆8-tetrahydrocannabinol (8-THC), ∆9-tetrahydrocannabinol (THC), ∆9-tetrahydrocannabinolic acid (THCA-A), cannabichromenic acid (CBCA), cannabidiol (CBD), cannabidiolic acid (CBDA), cannabigerol (CBG), cannabigerolic acid (CBGA), cannabinol (CBN) and ketoprofen (KET).

## Data Availability

The data presented in this study are available in article and Supplementary Materials.

## Supplementary Materials

The following supporting information can be downloaded at: https://www.mdpi.com/article/10.3390/molecules29122737/s1, Table S1. Inter-, intra-day and intermediate precision and accuracy.
