# Supplementary material for: Characterisation of Cannabis-Based Products Marketed for Medical and Non-Medical Use Purchased in Portugal
Source: Molecules. 2024 Jun 8;29(12):2737. doi: 10.3390/molecules29122737 (PMC11206000; doi:10.3390/molecules29122737)
Supplement: Supplementary file 1 [file molecules-29-02737-s001.zip › molecules-3015324-supplementary.pdf]

Supplementary Table S1. Inter-, intra-day and intermediate precision and accuracy.

		Beverage/oil					
		Inter-day (n=5)		Intra-day (n=6)		Intermediate (n=15)	
Analyte	Spiked (μg/mL)	CV (%)	RE (%)	CV (%)	RE (%)	CV (%)	RE (%)
CBD	0.4	5.52	2.77	3.00	-1.00	12.03	-6.21
	0.8	8.63	-0.56				
	1			6.60	-5.51	11.70	-6.09
	1.6	10.74	-0.28				
	3.1	7.89	-4.60				
	6.3	10.53	-3.83				
	10					5.52	-10.23
	12.5	5.12	-2.10				
	25	1.77	0.76				
	50	3.04	-1.34	3.37	-4.36		
	100	1.06	0.48	9.49	2.36	9.03	-3.85
CBDA	0.4	6.88	2.26	3.27	7.48	9.94	-2.47
	0.8	7.25	1.22				
	1			8.89	3.26	12.49	-3.58
	1.6	10.36	0.63				
	3.1	9.40	-6.42				
	6.3	9.85	-3.64				
	10					6.22	-9.60
	12.5	4.84	-1.83				
	25	2.70	1.55				
	50	3.53	0.43	3.38	-4.28		
	100	2.32	0.18	9.32	2.39	3.04	2.76
CBG	0.4	9.62	3.15	6.66	3.76	10.13	0.28
	0.8	8.72	1.78				
	1			6.73	-5.96	11.94	-4.15
	1.6	8.11	0.42				
	3.1	9.71	-6.31				
	6.3	10.81	-3.41				
	10					5.65	-6.29
	12.5	5.83	-1.83				
	25	8.72	5.48				
	50	2.68	-0.24	3.48	-4.35		
	100	1.90	0.80	6.54	-0.02	8.51	-1.30
CBN	0.1	12.21	2.62	4.56	10.17	7.79	7.24
	0.2	8.73	-2.07				
	0.4	6.62	-6.18	3.84	-12.36	9.96	-6.96
	0.8	4.86	-1.56				
	1			5.17	-9.21	11.49	-2.40
	1.6	9.63	0.78				
	3.1	11.15	-5.48				
	6.3	12.58	-2.01				
	10					6.62	-8.02
	12.5	5.67	0.51				
	25	2.49	3.34				
	50	1.65	1.59	3.34	-4.26		
	100	4.41	0.29	10.27	3.16	3.62	4.87
CBGA	0.4	8.28	3.29	3.19	9.88	12.00	0.99
	0.8	8.42	1.43				
	1			10.49	2.90	8.42	-0.94

	1.6	9.88	1.08				
	3.1	10.15	-6.03				
	6.3	9.87	-3.98				
	10					6.05	-6.15
	12.5	4.79	-1.57				
	25	2.72	1.99				
	50	3.21	0.54	3.41	-4.03		
	100	2.42	0.60	8.81	2.37	3.63	2.15
	0.4	10.40	2.17	5.93	2.72	14.16	-4.50
	0.8	9.51	-0.69				
	1			6.17	-8.90	14.67	-1.39
	1.6	8.37	-2.12				
	3.1	9.96	-5.02				
THC	6.3	11.19	-3.07				
	10					4.81	-7.78
	12.5	5.09	-0.16				
	25	2.98	2.52				
	50	0.90	1.47	3.75	-3.07		
	100	2.66	-0.12	9.29	3.10	10.90	-1.28
	0.4	8.93	4.48	5.83	-2.40	10.74	-4.63
	0.8	4.92	-0.18				
	1			6.04	-8.96	9.95	-5.77
	1.6	9.41	-0.30				
	3.1	9.65	-6.54				
8-THC	6.3	9.76	-2.40				
	10					4.88	-10.78
	12.5	4.25	-1.81				
	25	3.58	2.54				
	50	3.47	0.56	3.73	-3.32		
	100	2.21	1.29	9.14	2.87	8.71	-3.58
	0.4	17.02	-9.27	3.23	-12.56	15.51	-3.30
	0.8	8.48	-0.55				
	1			5.24	-7.94	10.35	-6.40
	1.6	11.52	0.34				
	3.1	6.44	-1.38				
	6.3	11.01	1.07				
THCA-A	10					1.91	-9.00
	12.5	8.79	1.06				
	25	7.01	4.48				
	50	7.60	3.30	3.35	3.48		
	100	5.63	6.00	5.06	7.52	3.54	3.03
	250	3.73	-1.96	8.15	-7.82	10.18	-1.37
	0.8	11.10	3.66	11.28	0.50	6.80	6.40
	1			11.10	3.24	13.30	-2.18
	1.6	6.86	0.58				
	3.1	7.06	-5.50				
	6.3	11.01	-2.45				
CBCA	10					8.02	-7.65
	12.5	4.77	-2.58				
	25	2.25	1.62				
	50	3.39	0.47	3.44	-5.12		
	100	3.34	1.80	7.29	0.43	3.23	1.83

Δ8-tetrahydrocannabinol (8-THC), Δ9-tetrahydrocannabinol (THC), Δ9-tetrahydrocannabinolic acid (THCA-A), cannabichromenic acid (CBCA), cannabidiol (CBD), cannabidiolic acid (CBDA), cannabigerol (CBG), cannabigerolic acid (CBGA), cannabinol (CBN), coefficient of variation (CV) and relative error (RE).

	Cosmetic products						
		Inter-day (n=5)		Intra-day (n=6)		Intermediate (n=15)	
Analyte	Spiked (μg/mg)	CV (%)	RE (%)	CV (%)	RE (%)	CV (%)	RE (%)
CBD	0.04	8.43	4.17	3.81	5.42	8.87	6.67
	0.08	2.63	-5.00				
	0.1			1.90	-2.70	2.46	-1.73
	0.16	2.11	-4.58				
	0.31	7.20	-3.55				
	0.63	6.77	-0.26				
	1					1.50	-4.63
	1.25	4.81	-2.21				
	2.5	3.93	-5.05				
	5	2.36	-5.55	2.70	5.37		
	10	1.80	7.08	4.57	3.08	3.07	5.83
CBDA	0.04	13.16	-0.17	5.44	-7.75	3.20	-5.83
	0.08	12.85	-2.08				
	0.1			7.01	-8.33	3.28	0.27
	0.16	6.15	3.12				
	0.31	4.57	4.73				
	0.63	3.70	1.27				
	1					2.15	7.63
	1.25	1.27	-3.12				
	2.5	7.36	2.16				
	5	2.09	9.18	5.22	9.52		
	10	5.81	-6.25	2.62	-3.23	2.91	8.67
CBG	0.04	7.20	11.67	3.64	5.75	4.16	5.42
	0.08	10.73	-5.83				
	0.1			4.28	7.00	1.49	2.33
	0.16	5.76	-2.08				
	0.31	7.48	-2.90				
	0.63	2.73	4.76				
	1					2.68	-0.53
	1.25	1.24	-0.11				
	2.5	2.65	11.33				
	5	5.23	-0.08	7.79	5.40		
	10	2.71	5.40	3.06	10.97	4.04	-5.00
CBN	0.01	10.11	-11.00	7.92	10.13	3.15	-8.33
	0.02	10.05	-0.33				
	0.04	4.00	-6.25	4.79	9.00	2.14	-4.83
	0.08	12.85	-2.08				
	0.1			1.14	-12.00	7.29	-8.67
	0.16	8.14	0.21				
	0.31	5.64	6.34				
	0.63	0.77	-2.17				
	1					7.19	6.57
	1.25	4.04	-4.72				
	2.5	6.57	-1.07				
	5	1.18	6.37	3.41	5.74		
	10	3.37	-3.82	1.64	-2.17	5.63	5.58
CBGA	0.04	3.58	6.67	7.39	-5.42	8.56	-6.67
	0.08	3.20	12.92				
	0.1			4.32	6.00	2.41	4.33
	0.16	5.35	1.87				
	0.31	6.32	-7.10				
	0.63	4.96	0.53				

	1					1.59	-3.20
	1.25	3.13	1.01				
	2.5	1.81	-2.07				
	5	2.05	-5.54	2.63	-4.10		
	10	5.52	4.89	4.95	4.63	4.14	4.03
	0.04	7.10	-13.25	2.58	-7.50	5.08	5.33
	0.08	5.72	3.33				
	0.1			1.16	-2.27	4.70	-3.88
	0.16	5.41	10.96				
	0.31	3.83	1.08				
THC	0.63	3.16	5.66				
	1					7.94	-0.47
	1.25	4.34	-3.60				
	2.5	7.50	-3.77				
	5	10.20	1.08	4.16	-5.13		
	10	3.81	1.57	0.63	-0.70	5.71	-1.70
	0.04	7.43	12.08	7.72	1.00	3.27	-7.42
	0.08	3.17	-11.04				
	0.1			5.18	6.33	4.00	9.00
	0.16	9.87	4.17				
	0.31	2.51	4.09				
8-THC	0.63	5.16	2.49				
	1					6.91	-8.50
	1.25	3.78	8.24				
	2.5	2.28	-2.39				
	5	2.93	-7.06	2.48	5.60		
	10	1.57	-2.09	5.46	1.03	7.61	-6.75
	0.04	14.13	-4.67	5.07	-9.50	4.46	7.08
	0.08	5.51	-8.33				
	0.1			5.85	-7.87	1.89	10.33
	0.16	5.42	4.58				
	0.31	4.66	4.84				
THCA-	0.63	4.31	1.64				
A	1					2.45	-1.83
	1.25	2.50	7.33				
	2.5	10.38	-1.15				
	5	1.45	8.29	4.59	2.66		
	10	2.58	1.82	4.66	0.02	4.76	3.72
	25	1.18	-6.34	8.49	5.12	1.47	0.48
	0.08	1.68	13.33	5.87	-12.00	2.99	-12.92
	0.1			2.34	7.80	2.57	3.00
	0.16	3.97	7.29				
	0.31	4.67	2.47				
	0.63	4.27	7.46				
CBCA	1					3.40	4.90
	1.25	6.11	0.51				
	2.5	12.12	2.19				
	5	2.84	10.03	3.67	5.94		
	10	9.36	0.26	3.35	2.43	1.12	-2.47

Δ8-tetrahydrocannabinol (8-THC), Δ9-tetrahydrocannabinol (THC), Δ9-tetrahydrocannabinolic acid (THCA-A), cannabichromenic acid (CBCA), cannabidiol (CBD), cannabidiolic acid (CBDA), cannabigerol (CBG), cannabigerolic acid (CBGA), cannabinol (CBN), coefficient of variation (CV) and relative error (RE).

		Herbal samples					
		Inter-day (n=5)		Intra-day (n=6)		Intermediate (n=15)	
Analyte	Spiked (μg/mg)	CV (%)	RE (%)	CV (%)	RE (%)	CV (%)	RE (%)
CBD	0.04	10.35	-6.00	11.63	-8.75	4.71	-12.50
	0.08	11.43	4.25				
	0.1			10.74	-8.00	7.20	-14.60
	0.16	7.37	-3.00				
	0.31	2.89	-1.32				
	0.63	1.47	3.92				
	1					11.42	4.04
	1.25	4.91	-2.64				
	2.5	4.02	-1.54				
	5	2.16	-3.33	1.65	-3.90		
	10	1.93	-2.30	1.97	-2.75	2.13	-2.70
CBDA	0.04	8.49	-8.25	9.55	-10.00	6.31	-2.50
	0.08	6.33	-4.38				
	0.1			11.39	-14.50	10.19	-13.20
	0.16	8.81	-3.50				
	0.31	2.85	-2.00				
	0.63	1.45	-1.35				
	1					9.52	-3.50
	1.25	5.72	-1.46				
	2.5	3.92	-3.80				
	5	5.74	-5.96	6.86	-6.36		
	10	3.05	-0.28	3.62	-0.15	1.81	-0.04
CBG	0.04	10.61	-8.75	9.77	-11.25	12.42	-9.00
	0.08	7.75	2.00				
	0.1			12.22	-11.50	9.55	-13.20
	0.16	7.77	2.19				
	0.31	4.08	-5.55				
	0.63	1.49	-2.22				
	1					8.32	-13.92
	1.25	4.82	-5.68				
	2.5	4.18	-3.60				
	5	2.66	-5.08	2.32	-5.00		
	10	1.97	-1.76	1.50	-2.10	2.71	-2.48
CBN	0.01	9.52	-2.30	10.49	-4.00	8.94	-6.80
	0.02	11.38	6.50				
	0.04	5.41	-6.00	5.16	-7.50	4.61	-8.50
	0.08	6.46	-4.88				
	0.1			2.31	4.00	4.23	4.00
	0.16	3.58	-4.75				
	0.31	6.68	-6.00				
	0.63	1.34	-1.27				
	1					5.49	-9.80
	1.25	4.28	-0.27				
	2.5	3.35	-3.96				
	5	2.42	-2.73	2.51	-3.70		
	10	1.07	-1.44	1.06	-1.88	1.68	-1.21
CBGA	0.04	10.47	-2.25	11.02	-3.75	12.03	-7.00
	0.08	5.51	-6.75				
	0.1			10.52	-11.50	9.63	-3.80
	0.16	3.82	-5.12				

	0.31	4.27	-5.35				
	0.63	2.76	-1.43				
	1					6.29	-5.80
	1.25	2.81	-3.46				
	2.5	3.26	-1.40				
	5	3.21	-3.72	3.18	-4.40		
	10	2.29	-1.67	2.37	-1.70	3.12	-1.44
	0.04	9.73	-0.75	11.22	-1.25	8.64	1.00
	0.08	3.42	-9.87				
	0.1			11.39	-2.50	5.84	-6.60
	0.16	3.64	-2.12				
	0.31	5.51	1.35				
THC	0.63	1.15	-1.83				
	1					8.74	-9.00
	1.25	3.48	2.98				
	2.5	4.25	-2.52				
	5	2.71	-0.68	3.14	-0.90		
	10	2.07	-2.30	2.37	-2.00	1.48	-2.80
	0.04	4.23	4.75	3.44	6.25	2.38	6.00
	0.08	9.93	0.63				
	0.1			7.56	-6.00	1.24	-8.20
	0.16	4.59	-3.62				
	0.31	2.80	-1.48				
8-THC	0.63	2.08	-2.62				
	1					6.99	-4.40
	1.25	4.29	-2.27				
	2.5	3.92	2.12				
	5	9.63	-5.28	3.84	-7.60		
	10	2.61	-3.48	2.19	-4.30	1.98	-4.56
	0.04	6.54	1.50	7.55	2.50	2.34	4.50
	0.08	4.79	-4.62				
	0.1			11.87	-2.15	8.79	-1.06
	0.16	4.83	0.38				
	0.31	3.11	-5.47				
THCA-A	0.63	1.82	-1.38				
	1					6.48	-7.82
	1.25	1.74	0.66				
	2.5	7.41	1.90				
	5	3.39	-6.46	3.33	-7.80		
	10	1.54	-2.41	1.46	-2.80	1.90	-2.70
	25	1.00	-1.65	0.41	-1.70	1.81	-1.50
	0.08	6.55	-4.25	7.66	-6.87	2.54	-4.50
	0.1			10.50	-3.00	0.44	-2.60
	0.16	5.83	10.50				
	0.31	3.40	-4.52				
CBCA	0.63	1.20	-1.17				
	1					4.49	-6.12
	1.25	3.71	0.92				
	2.5	2.38	-0.19				
	5	2.70	-4.48	1.66	-5.50		
	10	2.05	-2.73	1.58	-3.55	1.30	-3.46

Δ8-tetrahydrocannabinol (8-THC), Δ9-tetrahydrocannabinol (THC), Δ9-tetrahydrocannabinolic acid (THCA-A), cannabichromenic acid (CBCA), cannabidiol (CBD), cannabidiolic acid (CBDA), cannabigerol (CBG), cannabigerolic acid (CBGA), cannabinol (CBN), coefficient of variation (CV) and relative error (RE).
